# Illuminating the dark side of the human transcriptome with long read transcript sequencing

**DOI:** 10.1186/s12864-020-07123-7

**Published:** 2020-10-30

**Authors:** Richard I. Kuo, Yuanyuan Cheng, Runxuan Zhang, John W. S. Brown, Jacqueline Smith, Alan L. Archibald, David W. Burt

**Affiliations:** 1 The Roslin Institute and Royal (Dick) School of Veterinary Studies, University of Edinburgh, Midlothian, EH25 9RG UK; 2 The University of Queensland, St. Lucia, Brisbane, QLD 4072 Australia; 3 School of Life and Environmental Sciences, University of Sydney, Sydney, New South Wales Australia; 4 Information and Computational Sciences, The James Hutton Institute, Invergowrie, Dundee, Scotland, UK; 5 Plant Sciences Division, School of Life Sciences, University of Dundee, Invergowrie, Dundee, Scotland, UK; 6 Cell and Molecular Sciences, The James Hutton Institute, Invergowrie, Dundee, Scotland, UK

**Keywords:** Human, Transcriptome, Long read RNA sequencing, Iso-Seq, TAMA, Annotation, Pacbio, Nanopore, Gene models, Bioinformatics

## Abstract

**Background:**

The human transcriptome annotation is regarded as one of the most complete of any eukaryotic species. However, limitations in sequencing technologies have biased the annotation toward multi-exonic protein coding genes. Accurate high-throughput long read transcript sequencing can now provide additional evidence for rare transcripts and genes such as mono-exonic and non-coding genes that were previously either undetectable or impossible to differentiate from sequencing noise.

**Results:**

We developed the Transcriptome Annotation by Modular Algorithms (TAMA) software to leverage the power of long read transcript sequencing and address the issues with current data processing pipelines. TAMA achieved high sensitivity and precision for gene and transcript model predictions in both reference guided and unguided approaches in our benchmark tests using simulated Pacific Biosciences (PacBio) and Nanopore sequencing data and real PacBio datasets. By analyzing PacBio Sequel II Iso-Seq sequencing data of the Universal Human Reference RNA (UHRR) using TAMA and other commonly used tools, we found that the convention of using alignment identity to measure error correction performance does not reflect actual gain in accuracy of predicted transcript models. In addition, inter-read error correction can cause major changes to read mapping, resulting in potentially over 6 K erroneous gene model predictions in the Iso-Seq based human genome annotation. Using TAMA’s genome assembly based error correction and gene feature evidence, we predicted 2566 putative novel non-coding genes and 1557 putative novel protein coding gene models.

**Conclusions:**

Long read transcript sequencing data has the power to identify novel genes within the highly annotated human genome. The use of parameter tuning and extensive output information of the TAMA software package allows for in depth exploration of eukaryotic transcriptomes. We have found long read data based evidence for thousands of unannotated genes within the human genome. More development in sequencing library preparation and data processing are required for differentiating sequencing noise from real genes in long read RNA sequencing data.

## Background

**Fig. 1:**
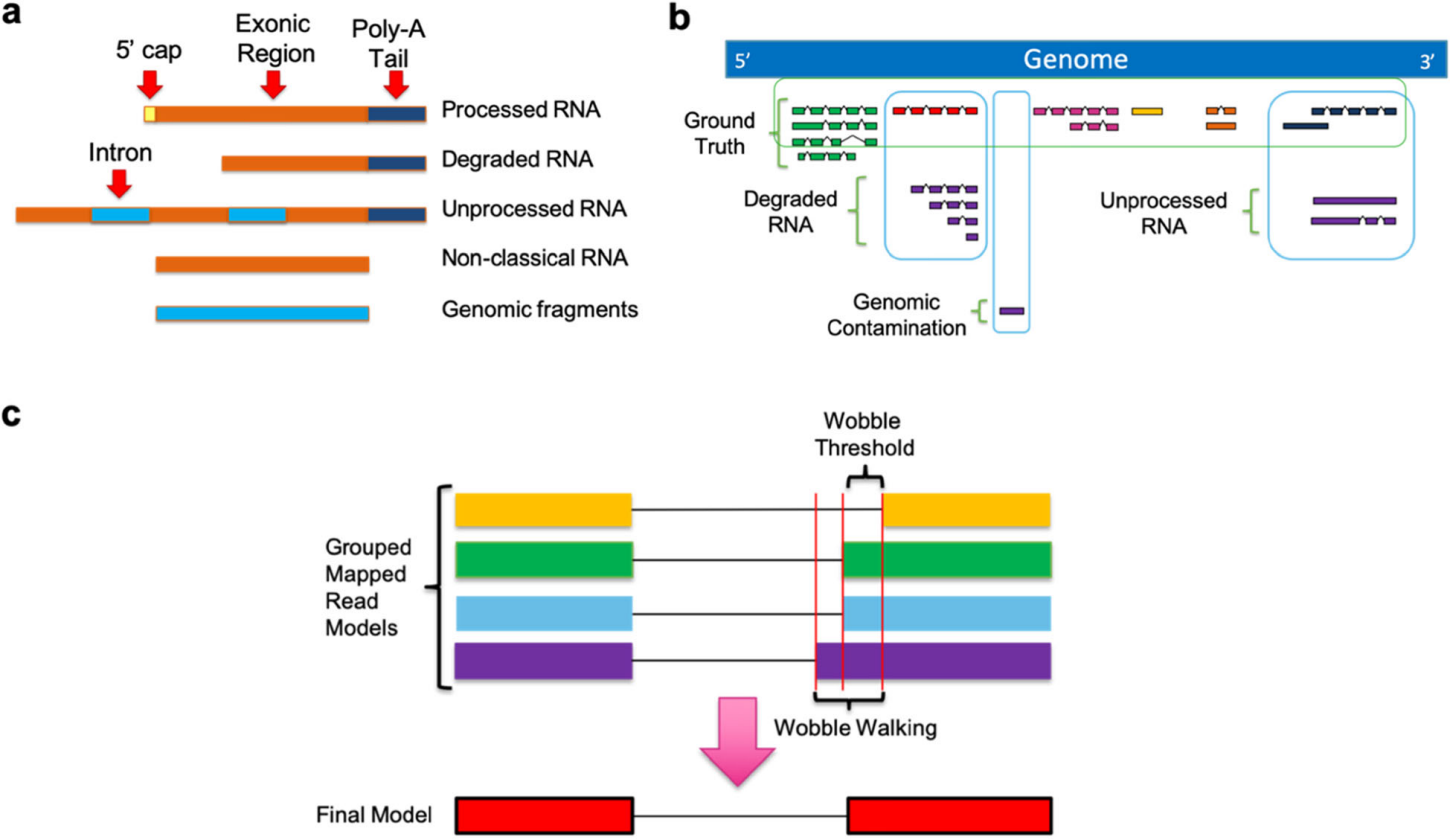
Long read RNA diversity and splice junction wobble. **a** RNA samples are typically comprised of a mixture of degraded and immature RNA as well as DNA fragments that can be erroneously identified as novel genes and transcripts. Non-classical RNA here represents lncRNA that do not have a 5′ cap or poly-A tail. **b** Representation of how RNA sample noise appears when mapped to the genome. **c** Due to sequencing errors, mapping reads can introduce wobble in determination of splice junctions. Wobble is defined as the difference between exon starts/ends between mapped reads. Wobble walking occurs when 3 or more transcript models have exon starts/ends with each closest pair occuring within the wobble threshold but with the outer pair of exon starts/ends having an in between distance greater than the wobble threshold. TAMA Collapse uses several methods of analyzing wobble to identify true splice junctions

The transcriptome remains a vastly underexplored space despite its significance as a foundation for biology. Major challenges for transcriptome annotation of eukaryotic species stem from biological complexity, RNA preparation, limitations of sequencing technologies, and sequence analysis. The biological complexity of alternative transcription start/stop sites and splice junctions [1] results in a combinatorial array of transcript sequences [2]. To complicate matters, RNA samples collected from eukaryotic species contain a mixture of mature functional RNA as well as pre-processed RNA, degraded RNA, and possible genomic contamination [3] (Fig. 1a-b). Meanwhile, low-throughput cDNA sequencing fails to provide coverage for rare/unstable transcripts, while short read RNA sequencing (RNA-seq) present computational challenges in accurate transcript model reconstruction [4–6]. The ambiguities created by these combined factors forced previous annotation software to adopt conservative algorithms that filtered out many real transcripts/genes such as single exon genes and long non-coding RNA (lncRNA).

High-throughput long read transcript sequencing provides higher confidence in predicting alternative transcripts and distinguishing real genes from sequencing noise [5]. While there have been many studies using long read transcript sequencing for transcriptome discovery [7–11], their sensitivity may have been compromised by the use of orthogonal verification/filtering. Filtering transcript models based on orthogonal information, such as requiring gene models to have sequence homology to annotated genes from closely related species, reduces gene discovery and is only applicable for a small number of species where such information exists [6].

The use of inter-read error correction in previous studies by either hybrid approaches (aligning short reads to long reads) or long read methods (aligning long reads into clusters) could also cause issues with both reducing gene detection sensitivity and producing erroneous gene models. Long read inter-read error correction methods such as PacBio’s Cluster/Polish method [7] filter out any reads that do not cluster with at least one other read. Due to the lower read depth of long read sequencing methods (relative to short read sequencing), this results in the removal of many low expressed genes and transcripts. Inter-read error correction methods can also produce erroneous hybrid sequences since long reads with higher error rates have an increased probability of grouping with other high error rate reads from different transcripts either from the same or paralogous genes. This type of error occurs when the alignment of reads is compromised by regions of high error density. While this effect could be reduced by requiring high alignment scores for clustering reads, this would also decrease the intended effect of rescuing low quality reads.

To leverage the power of long read transcript sequencing and address the issues with current processing pipelines, we developed the Transcriptome Annotation by Modular Algorithms (TAMA) tool kit. TAMA uses long read transcript data and high-quality reference genome assemblies to produce accurate and informative transcript models. TAMA is designed to improve transcript model prediction accuracy and increase transcriptome discovery with transparent and traceable steps. Evidence, including raw reads, read counts, local sequencing characteristics (e.g. mismatches, internal poly-A sequencing) supporting or compromising each transcript model is recorded and presented. This makes TAMA useful for situations where additional types of data, such as public annotations or short read RNA-seq, are not available [8]. In addition, by not relying on orthogonal information and having transparent and traceable steps, TAMA also provides a more agnostic approach to transcriptome annotation which can reveal problems with prior assumptions from previous annotation efforts.

We report the use of TAMA to analyze the Universal Human Reference RNA (UHRR) Sequel II Iso-Seq data released to the public by Pacific Biosciences (PacBio). This dataset represents the combination of the highest read depth for long read sequencing on a single human RNA sample with the highest long read accuracy. As such, the challenges of analyzing this dataset are applicable to all long read transcriptome datasets. We compared different long read based transcriptome assembly methods to identify corresponding benefits and issues. Our analyses indicate that long read transcript sequence data together with appropriate analysis tools has the potential to reveal yet further complexity in eukaryote transcriptomes.

## Results

### TAMA – Transcriptome annotation by modular algorithms

TAMA is comprised of modular tools with transparent algorithms, precise parameter control, and traceable outputs to allow users to analyze, interpret, and diagnose the resulting transcript models. The main analysis functions consist of two modules: TAMA Collapse and TAMA Merge.

TAMA Collapse uses mapped reads and a reference genome assembly to create a transcriptome annotation. TAMA Collapse uses four main methods for identifying true splice junctions: alignment quality filtration, local density error filtration (LDE), splice junction ranking, and splice junction coverage. All of these methods can be tuned by the user. First, alignment quality filtration is applied by assessing the alignment length coverage and alignment identity of each mapped read with respect to the reference genome. Reads below the user defined thresholds are discarded. The reads passing this first step are then examined via the LDE algorithm for the number of mismatches flanking each predicted splice junction. Errors around splice junctions exacerbate mis-mapping and cause the prediction of false splice junctions. This assessment removes reads with high error density within a specified base pair distance from each splice junction. The remaining reads are then grouped based on exon-intron structure allowing for user defined differences (called wobble in the TAMA nomenclature) in exon starts and ends measured in base pairs (Fig. 1c). The predicted splice junctions for the grouped reads are then ranked based on the flanking mismatch profiles and coverage. The highest ranked splice junctions are then used in the final transcript model. A large wobble threshold can help remove false positive predictions for splice junctions but may remove real splice junctions within the wobble length. Thus the LDE algorithm and splice junction ranking allows for smaller wobble lengths while also reducing false splice junction predictions.

In addition to rigorously identifying splice junctions, TAMA Collapse also allows the incorporation of the confidence of transcript starting sites by running the program in a capped or non-capped mode. For example, for 5′ captured RNAs, the capped mode will allow the transcripts with alternative transcript starting sites to be retained; while for non 5′ captured RNAs, the non-capped mode removes transcript models which appear to be 5′ degraded. The capped mode, requires grouped mapped reads to have the same number of exons and the same exon-intron structure. The non-capped mode is similar to the capped mode but allows for grouped reads to have differences in the number of exons on the 5′ end reflecting reads derived from RNAs with degradation from the 5′ end. Thus, all predicted splice junctions for the shorter mapped read model and the 3′ end would have to match those of the longer model. These two methods of grouping are described in a previous study where they were referred to as Transcription Start Site Collapse (equivalent to capped mode) and Exon Cascade Collapse (equivalent to non-capped mode) [4].

In addition to the transcriptome assembly, TAMA Collapse also outputs detailed information showing read mapping quality, collapsed read groups, predicted sequence variation, and transcript models with 3′ genomic poly-A (genomic contamination or truncated transcript). These outputs are intended to provide users with a full understanding of the behavior of TAMA Collapse and thus allow users to trace, diagnose, and improve their transcriptome assemblies.

TAMA Merge combines transcript models by examining exon-intron structures of transcript models to create a non-redundant set of genes and transcripts. TAMA Merge can be used on a single input transcriptome annotation to remove redundancy or can be used on multiple transcriptome annotations to create a unified annotation. TAMA Merge also produces output files that can be used to understand the differences between the input annotations. TAMA Merge uses the same collapsing mode algorithms from TAMA Collapse. One unique feature of TAMA Merge is the ability to merge transcript assemblies by assigning different collapsing modes and transcript model feature priorities between different annotations. For example, when using TAMA Merge to combine a long read sequencing derived annotation to a reference annotation, the reference annotation can be given priority for transcription start/end sites and splice junctions. The user created annotation can also be set to the non-capped mode to allow user produced models to collapse with 5′ longer reference models. The output files from TAMA Merge include detailed reports on how merging was done. These report files show which input annotations supported each of the final transcript and gene models as well as the amount of wobble that occurred at each exon start and end between merged models.

Along with TAMA Collapse and TAMA Merge, the TAMA toolkit contains many other tools that either apply additional filters or add information. Other TAMA tools used in this study are explained in further detail in the Methods section. A more detailed description of how TAMA works can be found here: github.com/GenomeRIK/tama/wiki/.

### Benchmarking TAMA and related software

We benchmarked the long read based transcriptome assembly of TAMA, Stringtie2 [9], TALON [10], and Cupcake [7] using three different datasets: simulated PacBio data, simulated Nanopore data, and PacBio Sequel II Iso-Seq data from Lexogen’s Spike-in RNA Variant (SIRV) control mix. The simulated PacBio and Nanopore reads were produced in a previous study [11] using PBSIM [12] and were also used for benchmarking in the Stringtie2 study [9]. The simulated datasets were based on the annotations of chromosome 19 of the human reference annotation. Details of the simulated and human datasets can be found in the supplementary files (Table S1). Using these simulated datasets, the Stringtie2 study showed that Stringtie2 outperformed both FLAIR [13] and Traphlor [14]. We used the same method of assessment as was used in the Stringtie2 study. While these simulated datasets are useful due to having a ground truth, they are not entirely accurate in their representation of long read sequencing data. In particular, the simulated reads were created by fragmenting transcript models at random which is not realistic since the fragmentation of transcripts is non-random and influenced by sequence characteristics and sample processing methods. The simulated PacBio dataset represents reads equivalent to PacBio Full Length Non-Chimeric (FLNC) reads. This means that they assume Circular Consensus Sequence (CCS) intra-read correction was performed and that adapters and poly-A tails were removed. The simulated Nanopore dataset is equivalent to Nanopore reads after removing poly-A tail and adapter sequences. Since PacBio’s Iso-Seq software (Cupcake) requires specific PacBio generated metadata that these simulated datasets do not contain, we could not benchmark PacBio’s Cupcake software on these datasets. This means that we could not use PacBio’s Cluster/Polish inter-read error correction on these datasets. Thus, these simulated datasets can only be used to assess the effect of random errors in long reads on the performance of mapping tools and transcriptome assemblies tools.

To address the issues with simulated datasets, we also used reads from the Lexogen SIRV spike-in from the PacBio UHRR Sequel II Iso-Seq dataset. The Lexogen SIRV control mix contains synthesized RNA molecules representing 7 expressed loci (18 genes when strand is accounted for) with 69 unique transcripts. The ground truth in this dataset is provided by Lexogen in the form of expected gene models based on their synthetic genome. However, it is possible that not all RNA from the SIRV dataset were sequenced and/or there are other RNA in the SIRV sample which are not represented in the annotation file provided on the Lexogen website. This may explain the lower precision of all unguided pipelines for the SIRV dataset (< 68% precision for all unguided approaches).

We used GffCompare [15] to calculate the sensitivity and precision for each pipeline. Sensitivity is defined as the number of correct transcript models in the predicted annotation divided by all the transcript models used for simulation. Precision is defined as the number of correct transcript models in the predicted annotation divided by the number of all predicted transcript models. These scores can be calculated at either the transcript or gene loci level. These definitions are from the GffCompare software. This method of calculation is identical to the method used in the Stringtie2 study [9]. Since TAMA, Stringtie2, and TALON can be run either with an unguided approach or a reference annotation guided approach, we tested both methods for each of these tools. Since TAMA is designed for parameter tuning, we applied two parameter sets for the unguided TAMA pipelines which we refer to as TAMA Low and TAMA High. TAMA Low uses parameters to maximize genic loci sensitivity at the cost of transcript model precision while TAMA High uses more stringent parameters to remove erroneous transcript models. The parameter selection for TAMA High and TAMA Low differs between the synthetic datasets and the PacBio Sequel II Iso-Seq data (SIRV and UHRR) since the synthetic datasets have higher error rates. TAMA High and TAMA Low parameter selection is described in more detail in the Methods section. Briefly, the TAMA High pipeline uses a more stringent LDE setting (fewer mismatches surrounding splice junctions), and requires read support from both SMRT Cells (in the PacBio Sequel II Iso-Seq data) while TAMA Low has lower stringency settings for LDE and requires support from only a single read. The TAMA High requirement of read support from both SMRT cells can be viewed as a modified form of the method that the Cluster/Polish step uses to filter out erroneous transcript models (removing all reads that do not cluster). However, the TAMA High approach can provide more sensitivity since it allows for greater variance on the 5′ end of the transcript models to account for low expressed genes which may only be represented by a 5′ truncated model in one of the SMRT cells (where the predicted 5′ complete model was picked up in the other SMRT cell). This method of filtration can also provide greater precision since requiring read support across sequencing runs can help reduce artifacts caused by technical batch effects. This algorithm can be adjusted where only a single SMRT cell or sequencing run was performed by only requiring multiple read support for each transcript model. This would still provide greater sensitivity than the Cluster/Polish method due to the greater allowance in 5′ variability. The TAMA Guided pipeline matches the transcript models from the long read data to the input reference annotation and adopts the splice junction predictions from the reference annotation. It discards any models not matching the reference annotation using the TAMA Merge algorithm. See Methods section for description of TAMA Merge and pipeline parameter selection.

**Fig. 2:**
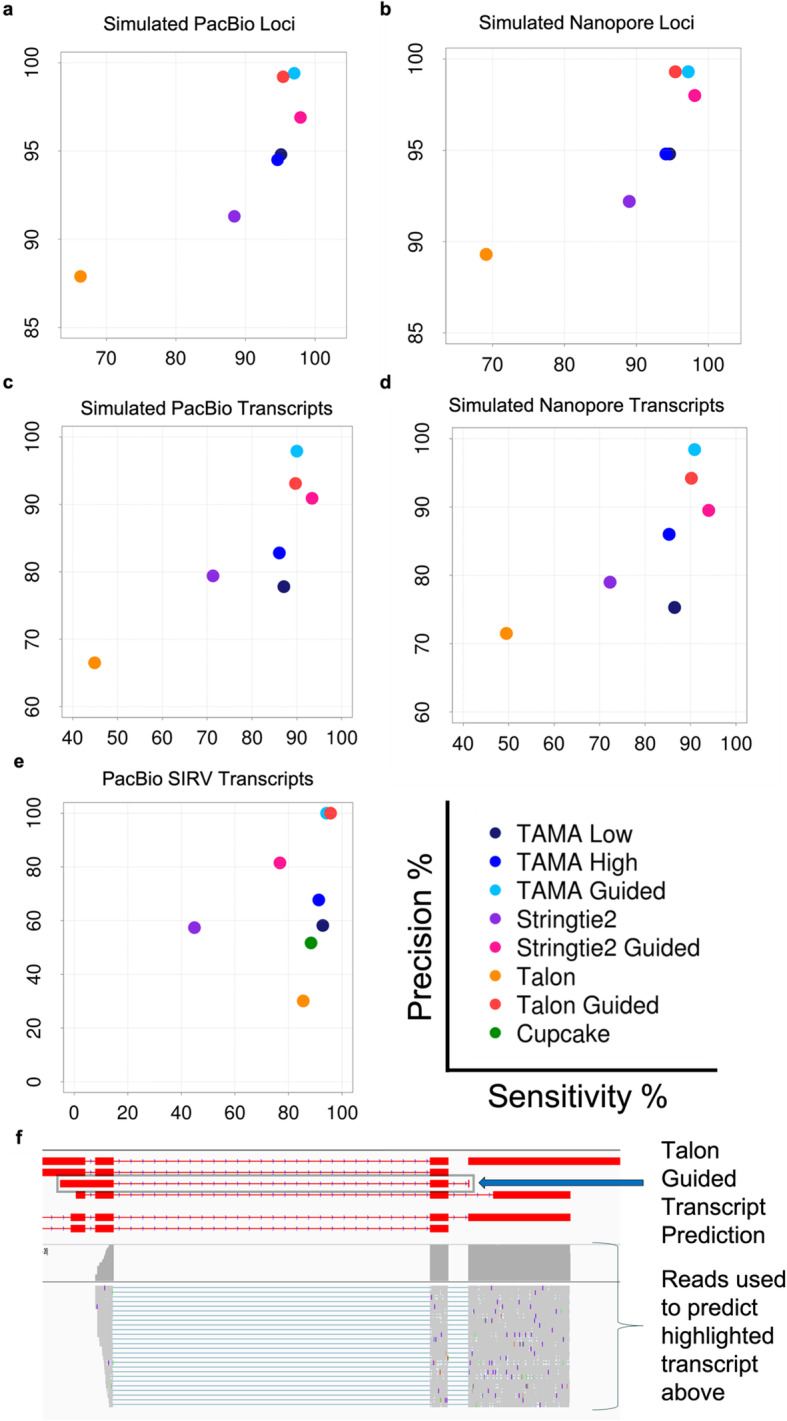
Long transcript assembly benchmarking. Sensitivity and precision of guided and unguided long read transcriptome assembly methods. **a** Gene loci level for simulated PacBio reads. **b** Gene loci level for simulated Nanopore reads. **c** Transcript level for simulated PacBio reads. **d** Transcript level for simulated Nanopore reads. **e** Transcript level for PacBio Sequel II Iso-Seq SIRV reads. **f** Example of erroneous transcript prediction by guided Talon where supporting reads are from another transcript model. These supporting reads are from 5′ degraded RNA resulting in the confusion

For both the PacBio and Nanopore simulated datasets, guided approaches achieved better sensitivity and precision as compared to unguided approaches (Fig. 2). The TAMA Guided approach had the highest precision across all datasets with slightly less sensitivity as compared to the Stringtie2 Guided approach for the simulated datasets. In the SIRV dataset, the TALON Guided method achieved a slightly higher sensitivity score as compared to TAMA Guided. The higher sensitivity score for TALON Guided was due to the inclusion of one more transcript model as compared to TAMA Guided. When we inspected this transcript model found only in the TALON Guided assembly, we found that it did not match the supporting reads (Fig. 2f). The reads used to support the TALON Guided prediction of that particular transcript model have a long 3′ extension as compared to the predicted transcript model. This extension is present in other transcript models in the SIRV annotation and it appears that these reads likely originated from 5′ truncated/degraded RNA from those transcripts. This raises the question of why these reads were assigned to the transcript model and how this might affect unguided TALON.

The overall better performance of guided approaches is to be expected because guided approaches essentially fit the transcript models to an annotation which has high similarity to the assessment annotation. However, guided approaches are not as useful for transcriptome discovery since they only confirm already known gene/transcript models. Among all the unguided methods, TAMA Low achieves the best sensitivity for the gene loci level while TAMA High achieves the highest precision and sensitivity at the transcript level compared to the non-TAMA approaches. The SIRV gene loci comparison was not included since the SIRV transcriptome is comprised of only 18 gene loci across 7 scaffolds. All methods had perfect sensitivity and precision at the gene loci level for the SIRV dataset.

### Effect of inter-read error correction on gene model discovery

**Fig. 3:**
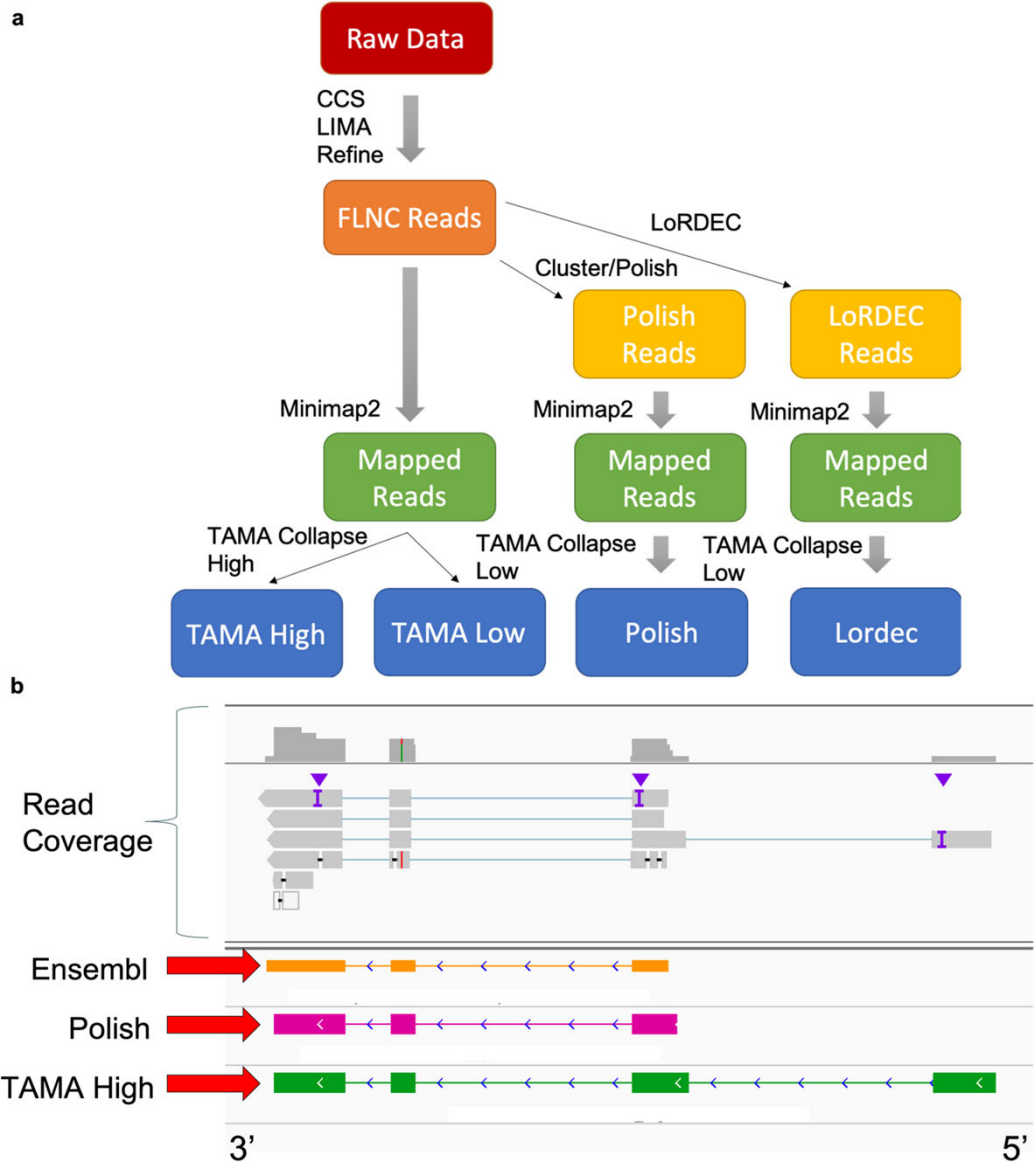
Comparing different pipelines on the UHRR Iso-Seq Dataset. **a** Diagram of workflow for the four pipelines used to analyze the UHRR Iso-Seq dataset. **b** Example of the Polish pipeline missing the full length transcript model due to low read coverage for the 5′ complete read. Since Cluster/Polish filters out any reads that do not cluster with at least one other read, the single read support for the longer model was filtered out in the Polish pipeline but captured by TAMA. In this case, the truncated model in Polish happens to match a transcript model in the Ensembl annotation

We processed the UHRR Iso-Seq data using four different pipelines to understand the effect of pre-mapping inter-read error correction on gene discovery and model prediction accuracy (Fig. 3a). The UHRR Iso-Seq dataset was comprised of two separate Sequel II runs using the 8 M SMRT Cells. There were 4,461,529 and 4,473,633 CCS reads generated by the two SMRT Cells which resulted in 3,504,905 and 3,447,471 FLNC reads, respectively. A plot of FLNC read lengths can be found in the supplementary files (Figure S1). All four pipelines use TAMA tools since the TAMA High pipeline has the highest combination of sensitivity and precision compared to all other non-guided methods in the benchmarking tests and the TAMA Low pipeline has the highest sensitivity. We compared two pipelines without inter-read error correction (TAMA Low and TAMA High pipelines), one pipeline using long read inter-read error correction (Polish Pipeline), and one pipeline using hybrid inter-read error correction (Lordec Pipeline). The Polish pipeline, uses inter-read error correction (in the form of clustering long reads and using the alignment to polish the sequences prior to mapping) along with TAMA Collapse using the same parameters as the TAMA Low pipeline. The Lordec pipeline, uses LoRDEC [16] inter-read error correction (aligning short read RNA-seq data to long reads prior to mapping) with TAMA Collapse (same settings as TAMA Low). For the Lordec pipeline we used short read RNA-seq data from the UHRR but from another study [17].

The TAMA Low and Lordec pipelines produced the most predicted gene and transcript models with more than 160 K genes and 750 K transcripts (Table 1). These extremely high numbers are likely due to issues with the use of reads with high error rates and reads originating from transcriptional noise. The Polish pipeline produced the fewest number of genes and transcript models (Table 1) while the TAMA High pipeline had over 1.5 times the number predicted genes but with a similar number of predicted transcripts.

**Table 1:** Pipeline comparison

Match type	Polish	Lordec	TAMA low	TAMA high
Total Genes	25,731	166,766	168,328	38,743
Total Transcripts	126,288	753,756	752,996	135,218
Ensembl Loci Overlap	19,348	30,835	30,947	21,284
Ensembl Transcript Matches	17,948	24,660	24,691	15,854
Predicted Novel Gene Loci	8519	139,769	141,097	23,302
Predicted Novel Transcripts	106,243	724,316	723,759	118,148

Comparison of gene and transcript numbers across pipelines broken down into different categories. Ensembl loci overlap refers to the number of Ensembl v94 annotation gene models that are overlapped on the same strand by gene models from each Iso-Seq annotation. Transcript matches refer to Ensembl v94 transcript models with identical exon-intron structures as transcript models in each Iso-Seq annotation. The Ensembl v94 human annotation consists of 58,735 gene loci and 206,601 unique transcript models. In some cases, multiple Ensembl gene loci are overlapped by a single Iso-Seq gene locus leading to the differences between matching loci and predicted novel loci

### Estimating gene model detection accuracy

While there is no ground truth for the human transcriptome, we used the Ensembl v94 (Release 94, October 2018) human genome reference annotation [18] as a reference to understand how our results compare to current annotations. We identified the number of gene loci and transcript models from the Ensembl annotation with representation from each pipeline. The TAMA Low and Lordec pipelines had the highest number of matches for both gene loci and transcript models indicating high sensitivity. However, given the high total numbers of genes and transcripts, the annotations from these pipelines likely contain many erroneous gene and transcript models. The TAMA High pipeline had more gene loci matches but slightly fewer transcript matches compared to the Polish pipeline. This means that there were more transcripts per gene in the Polish pipeline annotation (4.9:1) versus the TAMA High annotation (3.5:1). The higher ratio of transcripts to genes in the Polish pipeline, as compared to the TAMA High pipeline, suggests that either TAMA High is filtering out many real alternative transcripts or that Cluster/Polish is somehow predicting more erroneous alternative transcript models.

When we investigated the reason for the higher number of transcript model matches in the Polish annotation, we discovered that in some cases the Polish transcript models matched the models in the Ensembl annotation due the removal of reads (by the Cluster/Polish step) which supported 5′ longer transcript models (Fig. 3b). In these cases, the mapped reads showed 5′ extended transcript models with additional 5′ exons along with 5′ shorter models that may have originated from 5′ degraded RNA molecules. However, since the longer models had lower read coverage, the Polish pipeline removed them from the transcriptome assembly leaving only the shorter models that sometimes matched models in the Ensembl annotation. This tendency toward producing truncated transcript models could explain the expansion of alternative transcript predictions in the Polish pipeline. While it could be argued that these shorter models are real since they are represented in the Ensembl annotation, it is also possible that these RNA are typically rapidly degraded and thus full length representations have not been identified in the Ensembl annotation due to a lack of coverage from the supporting data used by the Ensembl pipelines.

### Assessing RNA degradation from Iso-Seq data

To gain a better understanding of the effect that RNA degradation may have on long read based annotations, we analyzed the transcript models which had matching 3′ exon-intron structure between the TAMA High (135,218 transcripts), Polish (126,288 transcripts), and Ensembl v94 (206,601 transcripts) annotations to see which annotation had longer 5′ representation (Table 2). When comparing the TAMA High annotation to the Polish annotation, there were 67,480 transcript models with matching 3′ exon-intron structure. Out of those 3′ matching transcript models, 56,198 (83.2%) showed the TAMA High models as having the longer 5′ representation with 3357 models (5%) having additional 5′ exons. This indicates that the Polish pipeline may be producing a large number of 5′ incomplete transcript models. While the TAMA High and Polish annotations had similar numbers of transcript models, roughly half of those models in each annotation did not have matches between the annotations. This may be due to differences in splice junction calls between the two pipelines which is referred to in this text as splice junction wobble.

**Table 2:** Comparing 5′ completeness of transcript models between annotations

Match comparison	TAMA high longer	Polish longer	Ensembl longer	Total matches
TAMA High - Polish	56,198	11,282	–	67,480
TAMA High - Ensembl	15,230	–	8312	23,542
Polish - Ensembl	–	15,496	10,690	26,186

When we compared the TAMA High annotation to the Ensembl annotation using the same method, we found 23,542 3′ exon-intron structure matching transcript models. Out of those matching models, 15,230 (64.7%) showed the TAMA High models as having the longer 5′ representation with 3521 models (15%) having additional 5′ exons. Comparing the Polish pipeline annotation to the Ensembl annotation using the same method, we found 26,186 3′ exon-intron structure matching transcript models. Out of those matching models, 15,496 (59.2%) showed the Polish models as having the longer 5′ representation. This could indicate that over three thousand Ensembl transcript models have incomplete 5′ ends with missing 5′ exons or that at least these represent novel alternative transcripts for these genes. Even though roughly half of the transcript models (67,480) from the TAMA High and Polish pipelines had matches between the two pipelines, less than half (23,542 for TAMA High and 26,186 for Polish) of those transcript models also matched the Ensembl annotation. This suggests that the models matching between the TAMA High and Polish pipelines but not found in the Ensembl annotation may represent novel alternative transcript models. Alternatively, they may indicate a type of systemic error in the transcript model prediction pipelines.

**Fig. 4:**
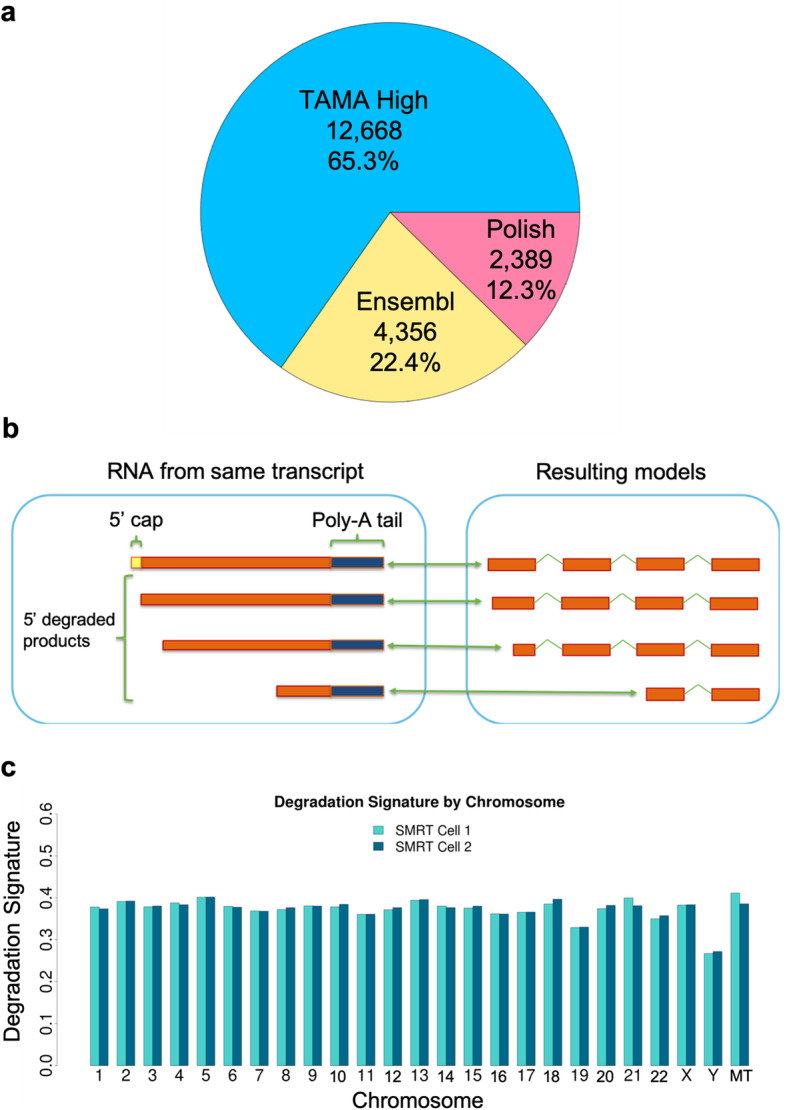
Degradation signature analysis. **a** Pie chart of the 3′ transcript level intersection between the TAMA High, Ensembl, and Polish annotations with the sections representing the number of 5′ extended transcript models from each annotation. **b** Diagram of degraded RNA representation with respect to a genome assembly. The reduced 5′ coverage results in 5′ variability in mapped reads. **c** Degradation signature by chromosome per SMRT Cell run

We then compared the intersection between all three annotations and identified 19,413 transcripts with common 3′ regions. Of these transcripts, TAMA High had the longest transcripts in 65.3% of the matches, Ensembl in 22.4%, and Polish in 12.3% (Fig. 4a). Although the Polish pipeline annotation had more 3′ matching transcript models with the Ensembl annotation in the two way comparison, the number of 5′ longer transcripts were similar to the TAMA High annotation suggesting that the increase in matches came from Polish pipeline models which were shorter on the 5′ end as compared to the matching Ensembl transcript models. While the 5′ shorter transcript models from the Polish pipeline may be accurate, these results demonstrate that the use of transcript model matching for assessing pipeline performance (as is used in GffCompare) can be affected by false positives from 5′ incomplete models where these models happen to match the reference annotation. Thus we suggest in depth evaluation of transcript models for a more accurate understanding of pipeline performance.

### A method for estimating RNA degradation from Iso-Seq data

To measure the relative amount of reads originating from 5′ degraded RNA, we developed a metric called the “Degradation Signature” (DegSig) which evaluates the amount of 5′ exon variability in transcript models (Fig. 4b). The DegSig metric is calculated using the outputs from TAMA Collapse runs and inputting them into the TAMA Degradation Signature tool. The value of DegSig is given as a percentage which represents the proportion of reads derived from 5′ degraded RNA (see Methods for formula). It is important to note that DegSig only provides an estimate of 5′ degradation with the caveat that bona fide alternative transcription start sites and incomplete first strand synthesis in the preparation of the cDNA library can also produce 5′ exon variability which can mimic 5′ degradation. To test our DegSig metric we applied it to two Iso-Seq datasets from Chicken brain RNA. One dataset was produced from TeloPrime [19] 5′ cap selected RNA and the other was produced without 5′ cap selection. The TeloPrime library should contain a lower percentage of degraded transcript sequences since it selects for complete capped RNAs. The non-cap selected data had a DegSig of 56.3% while the DegSig for the TeloPrime library data was 23.6%, suggesting a large difference in the proportion of degraded RNA sequences captured as cDNA by the two different methods. However, there is no ground truth in any species for the actual amount of 5′ shorter models with the same 3′ exon-intron structure as longer models, thus DegSig is only a rough gauge of the proportion of models which may be from degraded RNA.

We ran DegSig on the UHRR Iso-Seq dataset individually by SMRT cell and chromosome. Almost all chromosomes had a DegSig between 32 and 41% (Fig. 4c). However, the Y chromosome had a DegSig of 26.7 and 27.2% for SMRT Cell 1 and 2, respectively. One explanation for the much lower DegSig on the Y chromosome may be due to the lack of read depth for the Y chromosome (only 629 and 588 reads from SMRT cells 1 and 2, respectively). Lower read depths can decrease the DegSig values due to the lack of coverage for each gene. The range of DegSig for the human data is higher than that for the chicken 5′ cap selected RNA data, suggesting that there may be a significant number of reads from degraded RNA and thus reduced representation of full-length transcripts.

### Comparing splice junction identification accuracy

To understand the accuracy of each pipeline for predicting splice junctions, we looked at both mapping mismatch rates as well as splice junction wobble. Wobble refers to mis-mapping of splice junctions causing small differences in the genomic loci of mapped features such as exon boundaries and splice junction donor/acceptor sites (Fig. 1c) (See Methods for more detailed explanation of wobble). While the mismatch percentage of mapped reads are often used to assess the improvement of long read data from different error correction pipelines [20], this metric is actually not as useful for understanding the overall improvement in the transcriptome annotation. In genome-based transcriptome annotations, typically the most important features to identify are the transcription start sites (TSS), transcription end sites (TES), splice junctions, and exon chaining. These features allow for predictions of coding and promoter regions that are often crucial for downstream analyses. Thus, for transcript structure identification, errors near the splice junctions have a greater probability of altering the resulting transcript model than errors occurring farther away from the splice junctions. This means that the percentage of errors within a read may not be as impactful as the distribution of errors. Thus, another metric for the performance of error correction methods is to assess the amount of splice junction wobble between the predicted transcripts and known transcripts.

To demonstrate this concept we looked at the mapping mismatch profiles for each mapped read for the inter-read error correction pipelines (Polish and Lordec) and the pipelines using the mapped FLNC reads (TAMA High and TAMA Low). Note that the mapped FLNC reads are the same for the TAMA High and TAMA Low pipelines.

Using the output from TAMA Collapse we looked at length of mapped read coverage, mapping identity, clipping, insertions, deletions, and substitution errors. These values represent the comparison of the mapped reads to the genome assembly and thus only serve as an estimate of the true rates of error since difference between the reads and the reference genome assembly may be caused by real polymorphism. We calculated the average mismatch rates by counting the number of base pairs that were not matching between the mapped read and the genome sequence and dividing this number by the length of the mapped read. Mismatches evaluated include soft clipping, insertion, deletion, and substitution mismatches but do not include hard clipping.

**Fig. 5:**
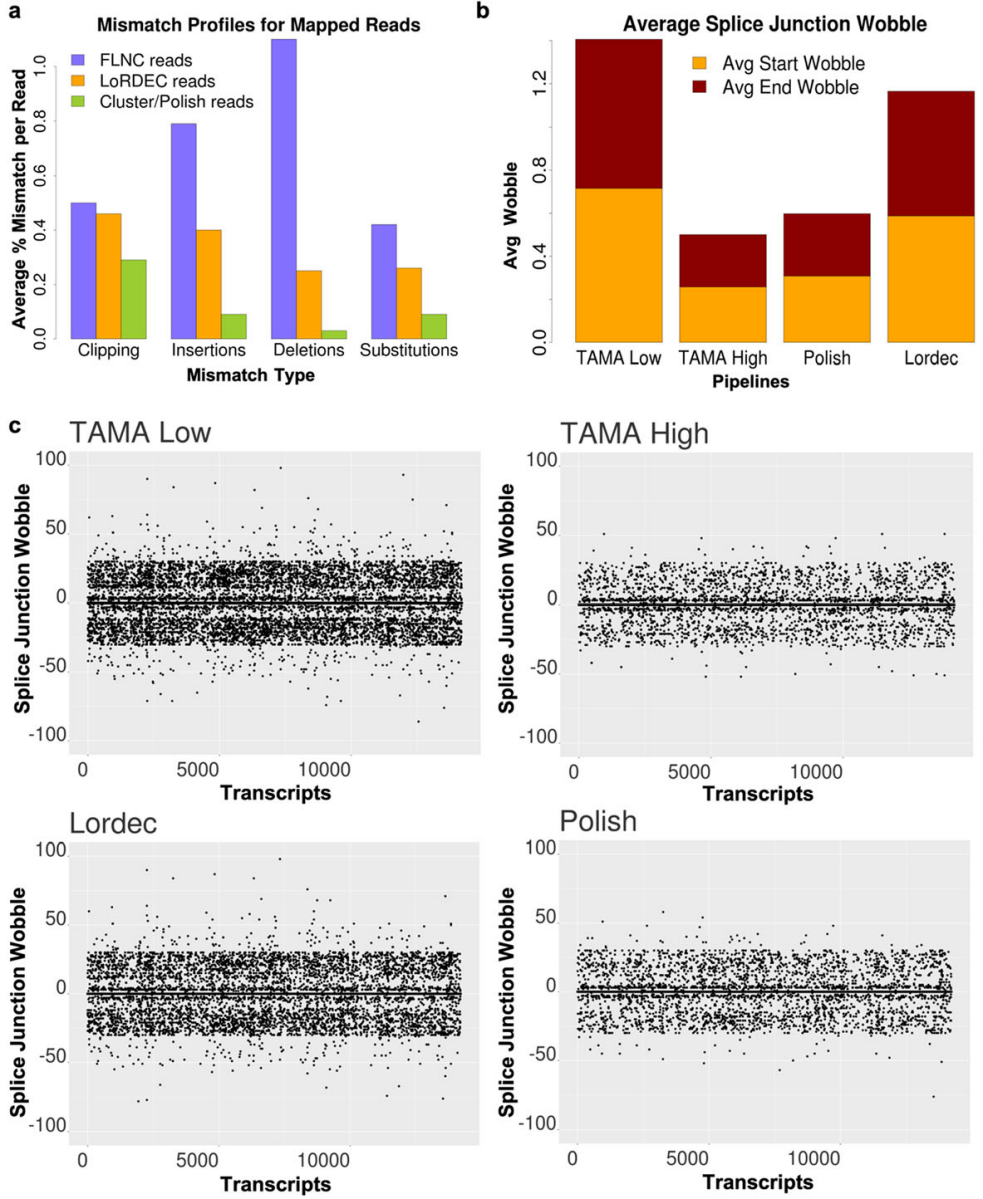
Error rate estimation and wobble across pipelines. **a** The average percent of alignment mismatch by mismatch type across pipelines. **b** Average splice junction wobble across all transcript models which matched the Ensembl annotation in all four pipelines. A splice junction wobble threshold of 30 bp on each side of the splice junction was allowed for matching for these plots. Note that wobble greater than 30 bp is possible due to wobble walking. **c** Scatter plots to illustrate the amount of wobble across all pipelines assessed on the transcript models used in Average Splice Junction Wobble plot

The mapped FLNC reads (used in TAMA High/Low pipelines) had the highest average predicted mismatch rate (2.83%) and the highest amount of each type of mismatch while the Cluster/Polish reads had the lowest mismatch rates (0.52%) with the lowest amount of each type of mismatch. The LoRDEC error corrected reads (average 1.38% mismatch rate) had a similar amount of clipping mismatches as compared to the mapped FLNC reads (Fig. 5a). This indicates that LoRDEC correction may have some issues correcting the ends of reads that may be due to lower short read coverage at the ends of transcripts.

We then looked at transcript model accuracy by measuring the wobble at splice junctions with respect to transcript models annotated in the Ensembl human annotation for the four different pipelines (Fig. 5b-c). Wobble typically occurs due a large number of read errors immediately flanking the splice junctions leading to small shifts in mapping the ends of each exon [21]. The total wobble for a splice junction within grouped reads can be larger than the specified wobble threshold due to a phenomenon we call wobble walking. Wobble walking occurs when the predicted exon starts/ends are represented in staggered formation so that the difference between each closest pair is still within the wobble threshold but the difference between the most distant pair is greater than the threshold (Fig. 1c). The amount of wobble between the transcript models of each pipeline compared to the reference annotation provides a metric for the accuracy of the transcript models produced by each pipeline. For instance, the expectation is that if a transcript model from a long read based annotation contains identical splice junctions (a splice junction wobble of zero) as compared to a reference annotation, then the long read based transcript model has the correct predicted splice junctions. We ignored wobble at the transcript start and end sites due to the high variance of these features in natural RNA [22, 23]. We also only assessed Ensembl transcript models that had coverage from all assessed pipelines to account for the differences in sensitivity between the pipelines.

The TAMA High pipeline with stringent LDE filtration had the lowest average wobble values per splice junction while the TAMA Low pipeline produced the highest average wobble (Fig. 5b-c). Thus, despite the lower overall error rates in the mapped reads from the Polish pipeline, the TAMA High pipeline had more splice junctions matching the Ensembl annotation. This suggests that the LDE filtration in the TAMA High pipeline resulted in more accurate identification of splice junctions.

### Inter-read error correction mis-clustering may produce erroneous gene models

One of the major concerns when using inter-read error correction methods such as Cluster/Polish and LoRDEC is the possibility of combining read sequences from different transcripts that would result in erroneous transcript models. The different transcripts could be from different genes (gene-level jumble) or a combination of alternative transcripts within the same gene (transcript-level jumble). Gene-level jumble typically occurs due to the sequence similarity of paralogues within gene families [23]. In both gene-level and transcript-level jumble, it is more likely that the highest expressed gene or transcript within the read clusters will mask the lower expressed genes. This is because the final cluster sequence is determined by sequence coverage. However, in cases where the read coverage within a jumble cluster is similar across unique transcripts, it is more likely that the resulting cluster read will have a mixture of sequences from each unique transcript within the cluster.

To investigate how often these jumble events occur, we compared the read mappings from the mapped FLNC reads (TAMA Low) to the inter-read error corrected reads (Polish and Lordec) to find reads that mapped to different genes and transcripts in each comparison. While it is possible that the FLNC read mappings are erroneous, they represent the read sequences without any over-correction. Also reads that map to different loci after inter-read error correction indicate that there is enough sequence ambiguity to call into question the effect of the inter-read error correction.

**Fig. 6:**
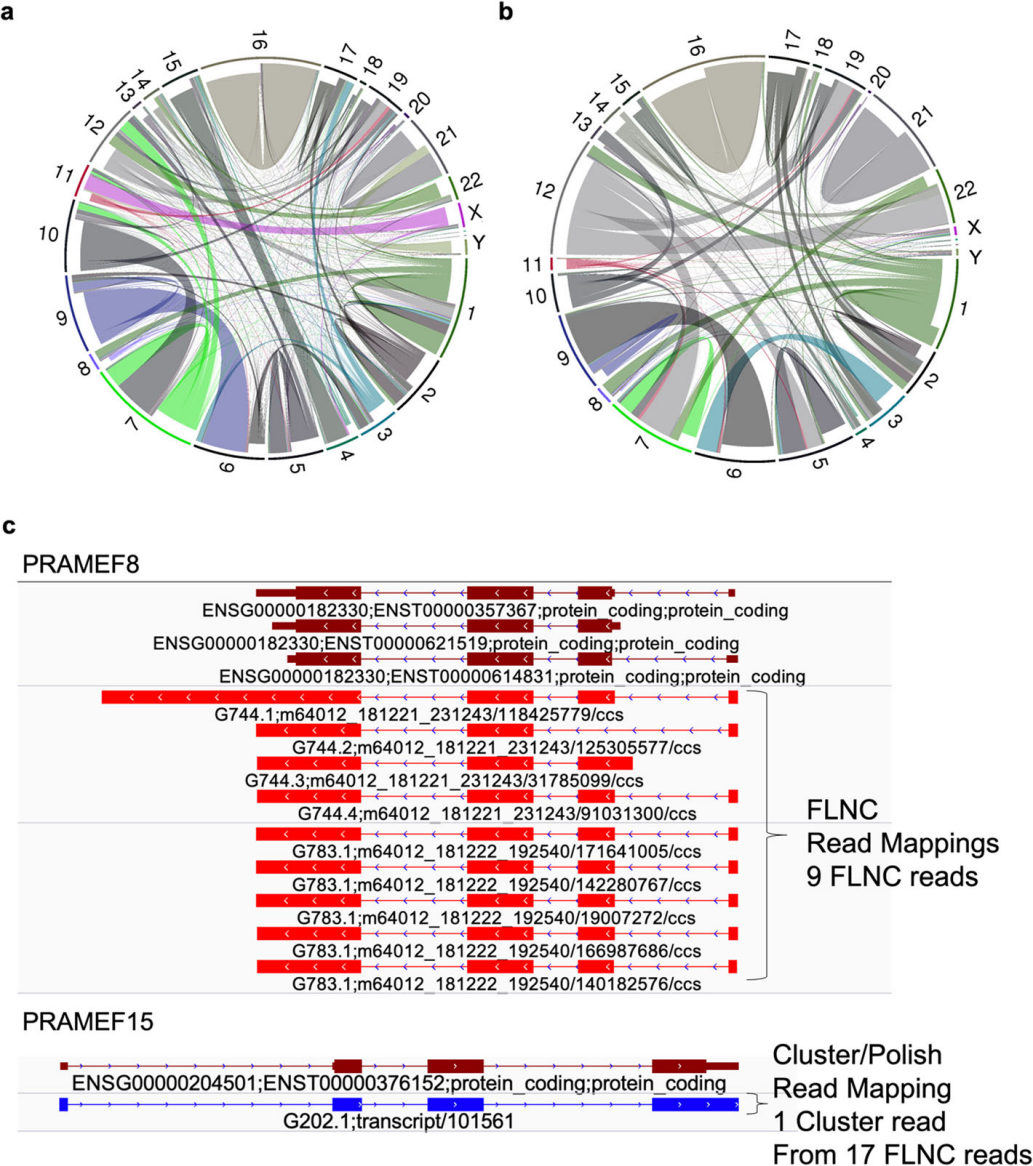
Gene and transcript read swapping from error correction. **a** Circos plot showing reads mapping to different loci after using Cluster/Polish for long inter-read error correction. Each line represents one read and the width of each chromosome bin represents the number of reads (combined thickness of each line). The indented line ends shows FLNC read location and non-indented ends shows read allocation after inter-read error correction. This plot shows 34,637 reads from 4799 genes moving to 2793 genes after Cluster/Polish. The reads are organized by chromosome however swapping occurs within chromosome and between chromosomes. **b** Circos plot as above but after hybrid inter-read correction with LoRDEC. Each line represents a single read moving from one gene to another with 19,064 reads from 2292 genes moving to 2319 genes after LoRDEC error correction. **c** The PRAMEF8 gene has coverage from 9 FLNC mapped reads (TAMA Low). Five of these reads were clustered and combined with other reads into one cluster read by Cluster/Polish resulting in a jumbled cluster read mapping to the PRAMEF15 gene (Polish pipeline). This suggests a false negative for PRAMEF8 and false positive for PRAMEF15 in the Polish pipeline due to the use of Cluster/Polish

Comparing the mapped FLNC reads to the Cluster/Polish mapped reads, we found 34,637 reads (0.6% of mapped reads) that switched from one gene locus to another after Cluster/Polish correction (Fig. 6a). This gene loci switching involved 6774 genes, 3230 of which were only found with the TAMA Low pipeline while 104 genes were only found with the Polish pipeline. The asymmetry of the number of unique genes between the pipelines suggests that Cluster/Polish may reduce gene discovery by combining reads from low expression genes with high expression genes.

To assess the effect of hybrid inter-read error correction on gene level read jumbling, we compared the mapped FLNC reads to the mapped LoRDEC corrected reads. There were 19,064 reads (0.3% of mapped reads) which switched from one gene locus to another (Fig. 6b), involving a total of 3476 genes, 775 of which were only found with the TAMA Low pipeline while 675 genes were only found with the Lordec pipeline.

To gain a more detailed understanding of what happens during a read jumble event, we examined the PReferentially expressed Antigen of MElanoma (PRAME) gene family. The PRAME gene family is highly associated with cancer development [24] and is used as a biomarker for identifying various forms of cancer. Within the PRAME gene family there are 24 annotated paralogues [25]. In this example, the Polish pipeline fails to detect one of the PRAME paralogues (PRAMEF8) while erroneously predicting the expression of another paralogue (PRAMEF15) which has no FLNC mapped read support. The TAMA Low pipeline (using FLNC mapped reads) finds 9 reads mapping to PRAMEF8 (Fig. 6c) while the Polish pipeline (using Cluster/Polish mapped reads) shows no reads mapping to PRAMEF8. Of the 9 PRAMEF8 reads from the TAMA Low pipeline, 5 of these reads were clustered and combined with other reads (3 from PRAMEF11, 4 from PRAMEF4, 2 from PRAMEF7, and 3 from PRAMEF27 according to FLNC mapping) into 1 cluster read by Cluster/Polish resulting in a jumbled cluster read mapping to the PRAMEF15 gene (Polish pipeline). We analyzed the sequence similarity between the two paralogues by aligning the PRAMEF8 and PRAMEF15 transcript sequences with Muscle [26] and found that they had 76% identity. While the two genes have similar exonic sequences, the genome mapping identity for the reads were higher than the sequence similarity between the two paralogues. The PRAMEF8 FLNC read with the lowest genome mapping identity score had a mapping identity of 89% and 6 PRAMEF8 FLNC reads had mapping identities over 98%. Thus, there is strong evidence that the reads mapped correctly in the TAMA Low pipeline and were altered to the point of mis-mapping in the Polish pipeline. This particular type of error could have major consequences for studies aimed at identifying gene biomarker expression.

We also examined how erroneous inter-read error correction can lead to transcript level jumbling. In this case, when reads from different transcripts from the same gene are grouped for error correction, the resulting sequence will, at best, represent only the more highly expressed transcript and, at worst, represent an erroneous jumbled sequence. Comparing the TAMA Low pipeline to the Polish pipeline, we found 477,351 reads that mapped to different transcript models within the same gene. There were 112,891 transcripts affected by transcript-level jumbling, 44,852 of which were found only in the TAMA Low annotation while 1372 transcript were found only in the Polish annotation. Comparing the TAMA Low pipeline to the Lordec pipeline, we found 187,829 reads that mapped to different transcript models. This involved 142,704 transcripts with 7117 transcripts found only in the TAMA Low annotation and 11,732 transcript found only in the Lordec annotation. It is important to note that this transcript level jumbling assessment is only a rough indication since without a ground truth for real transcripts it is impossible to know which transcript model is accurate.

To summarize, in both the long and short inter-read error correction pipelines we saw a significant number of gene-level and transcript-level read jumbling which may result in the prediction of gene and transcript models that are not biologically accurate. Hence, to avoid read jumbling issues we suggest foregoing inter-read error correction and instead focus on methods, such as the TAMA Collapse LDE algorithm, for removing reads with error profiles that could lead to erroneous transcript model predictions.

### Analysis of predicted expressed loci not found in the Ensembl human annotation

Given that the TAMA High pipeline had the highest sensitivity and precision scores for non-guided annotation in the benchmarking datasets, we used the gene loci predicted by the TAMA High pipeline to investigate potentially novel genes within the UHRR dataset. To gain insight into the 23,302 TAMA High predicted gene models not found in Ensembl (TAMA High specific gene models), we looked at several features which provide support for or against real gene models: coding potential, number of exons, intronic overlap with other genes, overlap with regulatory features, and the presence of immediately downstream genomic poly-A stretches. The combination of coding potential and splice junctions is often used as evidence of a functional gene. Conversely, overlap with introns (from other genes), genomic poly-A stretches immediately downstream of a gene model, and the absence of splice junctions (single exon transcripts) provide evidence that the source of the model could be from either non-functional transcribed products or genomic contamination.

Coding potential was assessed using three complementary methods. First, we used an open reading frame sequence analysis tool, CPAT [27], to detect coding potential. This method only works when the transcripts models do not contain frame shifts caused by erroneous splice junction calling. Second, we used TAMA merge to identify gene models that overlapped the genomic loci (on the same strand) of protein coding genes within the Ensembl annotation. Third, we used the TAMA ORF/NMD pipeline which is a frame shift-tolerant method of matching transcript sequences to peptide sequences from the UniProt [28] database. We combined these three methods to account for the various errors that can cause false negatives in protein coding gene prediction.

**Fig. 7:**
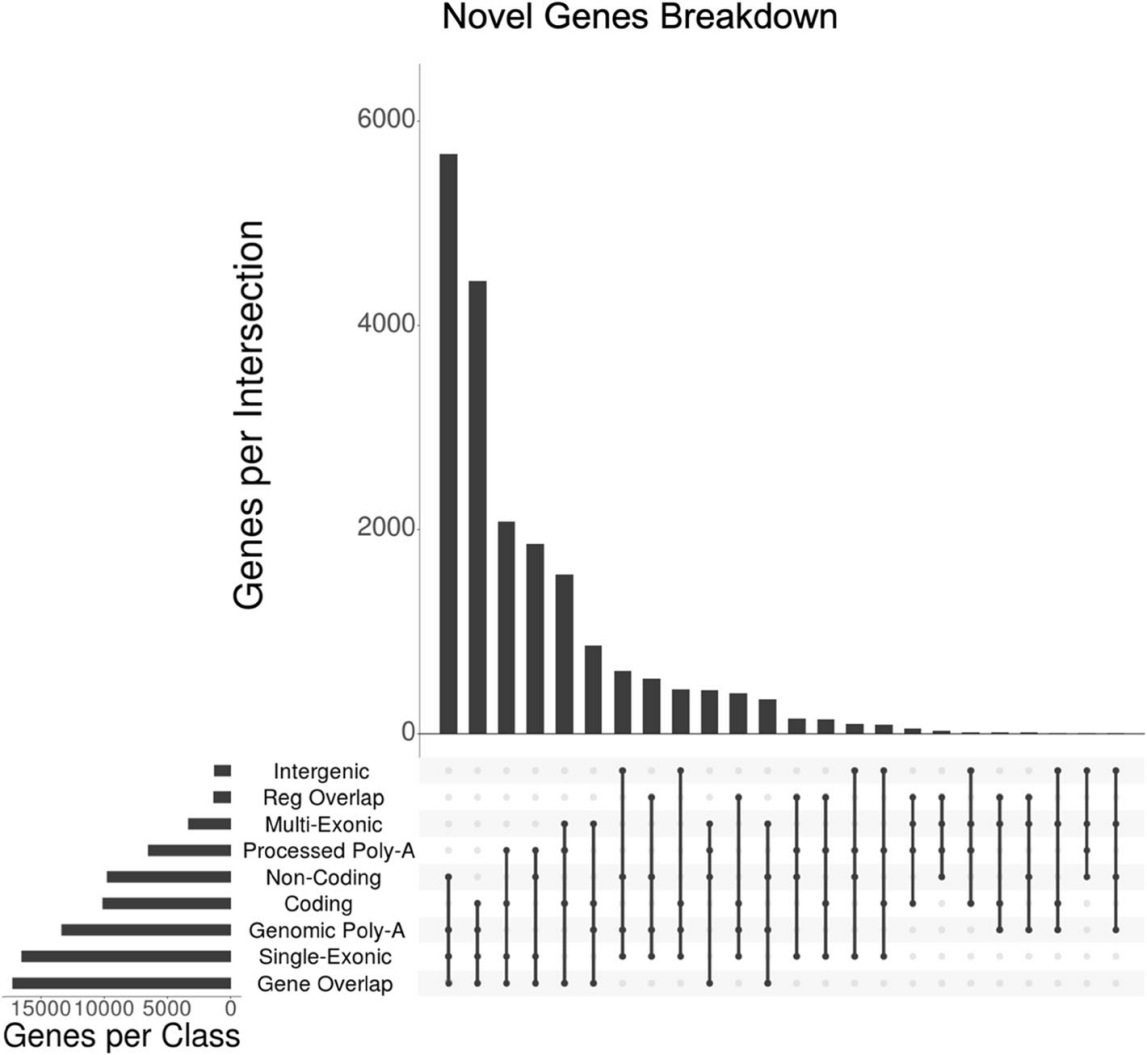
Putative novel genes breakdown. Novel gene breakdown by features. Combinations of features provide support for each gene being either real and belonging to a specific biotype or not real and the result of erroneous model predictions. The largest feature set faction are indicative of non-real models. However, there are still thousands of loci with feature sets which are compatible with real genes

Only a small number of the TAMA High predicted gene models which were not found in the Ensembl v94 annotation (18 out of 23,302) were supported by all features which are considered evidence for functionality (multi-exonic, coding, intergenic, and processed poly-A) (Fig. 7). This is expected given that these features are used by short read RNA-seq annotation pipelines for validation. Therefore, many of the gene models with these features are likely to have already been identified within the Ensembl annotation.

There were 1059 TAMA High specific gene models which were intergenic, single exonic, and had genomic poly-A. These features are commonly ascribed to genomic DNA contamination. However, the precise mechanism for how these sequences make it through to the final sequencing library is not well characterized.

The two most common sets of features for the TAMA High specific gene models are “single exonic, non-coding, intronic gene overlap, and genomic poly-A” at 24% (5679) and “single exonic, coding, intronic gene overlap, and genomic poly-A” at 19% (4440). These feature sets are typically used as indicators for non-real models since they could be derived from internal priming of unprocessed RNA. However, this would require further truncation of the template so that the resulting model does not overlap with transcripts from the gene of origin. In theory a subset of loci with the first feature set could be comprised of lncRNA while a subset of loci with the second feature set could be comprised of processed pseudogenes. Together, these account for over 43% of the TAMA High specific gene models.

There were 2566 (11% of TAMA High specific gene models) gene models that were predicted to be non-coding with processed poly-A tails. Of these, 461 were multi-exonic while 2105 were single exon genes (Fig. 7). Given that these models did not overlap any exonic regions of gene models in the Ensembl annotation, this would represent a large increase in the number of predicted lncRNA for the human genome.

There were 1557 (7%) TAMA High specific gene models with features (multi-exonic, coding, intron overlapping, and processed poly-A) that are indicative of real protein coding genes that exist within the introns of larger genes. However, it is possible that these are alternative transcripts from the surrounding genes but due to lack of 5′ completeness, the overlapping 5′ exons were not represented in the transcript models. If these gene models are derived from alternative transcripts of their surrounding genes, these models would represent novel transcripts.

These analyses were based on the Ensembl v94 human annotation, the Ensembl v100 annotation has since been released. This new Ensembl version has more than a thousand new lncRNA gene models as compared to v94. We compared the TAMA High annotation to v100 and found 144 matching lncRNA genes that were not present in v94. This raises questions regarding what exactly is present in our sequencing data and what is the best way to further dissect this information to produce biologically meaningful results.

With the UHRR being one of the most carefully prepared RNA samples, this would indicate that researchers would require more advanced methods of either RNA preparation and/or sequencing analysis to confidently identify novel genes.

## Discussion

The UHRR PacBio Sequel II Iso-Seq dataset is the result of one of the most accurate high-throughput long read transcript sequencing technologies [29] applied to an RNA library used as a reference for gene profiling experiments. Thus, this dataset represents the technological limits and challenges that are pertinent to all RNA sequencing studies as well as the potential of long read transcript sequencing for discovering novel genes and isoforms. To date, there has been a heavy emphasis on the use of multi-omics or orthogonal data to identify what is real and functional within the transcriptome. While this is certainly a powerful means of investigating novel genes, the pipelines developed for this purpose often overlook the need to properly process individual sources of data before integrating across data types. Using TAMA, we have demonstrated some key issues with current long read RNA sequencing data pipelines that could have a major influence on current transcriptomic studies. Firstly, mis-mapping of reads with sequence errors around splice junctions (error generated wobble) can produce transcript models with false splice junction predictions. Secondly, RNA degradation can result in 5′ incomplete transcript models that can have large downstream effects for data processing and interpretation. Thirdly, inter-read error correction can also cause false positives and negatives for gene and transcript model predictions. Finally, the combination of these problems also brings up challenges for using long read data in expression quantification experiments. If a significant number of reads can change transcript assignment due to either lack of 5′ completeness or changes in mapping loci after inter-read error correction, quantification estimates may not reflect the true biological state. While sequence error correction is currently the main focus of many long-read bioinformatic tools, it should not be applied at the cost of biological accuracy as could be the case for the gene and transcript read jumbling events from long read and short read inter-read error correction.

The resulting transcriptome annotation with TAMA portrays a very different composition of gene models compared to public transcriptome annotations. These differences suggest the existence of possibly thousands of potential novel genes (many of which are classified within under-represented biotypes) and/or artifacts arising during the sequencing pipeline.

The underlying issue in all methodologies is the balance between retaining useful information and discarding misleading information. However, the treatment of long read RNA data requires customization to accommodate both the sequencing technology as well as the biological assumptions. The TAMA tool kit is designed to allow the user to tune its behavior. This means that TAMA Collapse and TAMA Merge can be used with less stringent settings for maximum discovery potential and/or high stringent parameters for curating reference annotations. The resulting gene models can be assessed with the TAMA ORF/NMD pipeline for identifying coding similarity to know protein coding genes. However, more development is needed for discerning between long non-coding RNA and RNA sample noise. This may require wet lab methods such as improved 5′ cap selection for biasing against RNA sample noise.

From our analyses of the UHRR PacBio Sequel II Iso-Seq data with TAMA, we have identified that there are issues with RNA preparation methods and/or there are still thousands of novel genes that have not been annotated in the human genome.

## Conclusions

Long-read transcript sequencing presents new challenges for annotating transcriptomes. Analysis of the UHRR PacBio Sequel II Iso-Seq data suggests that there may be thousands of unannotated non-coding genes within the human genome. However, the methods for sequencing library preparation and data processing require more development to distinguish expressed genes from sequencing noise. Wobble analysis should complement read mapping identity for assessing transcript assembly pipelines. Degraded RNA within samples can lead to 5′ incomplete transcript model predictions. Inter-read error correction (prior to mapping) can cause read jumbling and gene model ambiguity. Read jumbling represents one challenge for using long read data for quantification. Long-read sequencing analysis benefits from tools (such as TAMA) which allow for algorithmic tuning to accommodate sequencing error rates and biological assumptions.

## Methods

TAMA Version Commit 39c1270c6e1ef2cf5d39f7f047fa15e0f1a6c790 was used for this study.

More detailed information on how TAMA works can be found here:

https://github.com/GenomeRIK/tama/wiki

### Wobble

Wobble is defined in this text as the distance measured in bases between the mapped starts and ends for exons. This term is used to describe small differences (< 50 bp) in predicted starts/ends based on mapped reads. These differences can occur due to real differences in starts/ends or due to errors in the reads flanking the starts/ends. For example, if a read has a number of missing bases immediately flanking a splice junction (SJ are comprised of one exon start and one exon end), the predicted splice junction from mapping may be off by the same number of missing bases. TAMA Collapse and TAMA Merge both use wobble to allow for the grouping of reads to be collapsed into a single transcript model. This is assessed by comparing every pair of transcript models within the same genomic loci (at least 1 bp same strand overlap connecting all loci grouped reads). In each pair assessment, each exon start and end from each predicted transcript model is compared to see if they occur within the user defined wobble threshold.

Due to this allowance of wobble between predicted starts and ends of exons, a phenomenon termed in this text as wobble walking can occur (Fig. 1c). Wobble walking is defined as a situation where 3 or more transcript models have exon starts or ends where the most upstream exon start/end prediction and the most downstream exon start/end prediction occur at a distance greater than the wobble threshold. However, the other exon start/end positions occur in such a way that when ordered based on genomic position there are no consecutive pairs of exon starts/ends which are farther apart than the wobble threshold. Thus by using the pairwise non-stochastic method of matching transcript models, all transcript models in this situation would match due to the linking effect across all represented exons starts/ends. When this situation occurs, the distance between the exon starts/ends between the grouped transcripts used for collapsing can be greater than the user defined wobble threshold.

### TAMA collapse

TAMA Collapse performs multiple functions: transcriptome assembly, variant calling, genomic downstream poly-A detection, and transcript/gene level quantification. The primary function is to create a non-redundant error corrected genome reference based transcriptome annotation. TAMA Collapse takes as input a sorted SAM/BAM file representing long read RNA sequencing data mapped onto a reference genome assembly as well as a fasta file representing the reference genome assembly used for mapping. TAMA Collapse is designed to be highly tunable and relies on 4 main parameters to define its behaviour: wobble thresholds, collapse mode, splice junction ranking, and the amount of mapping mismatch surrounding splice junctions (LDE).

The wobble thresholds and collapsing modes are used to define how mapped reads are grouped for collapsing. Wobble thresholds can be defined for the TSS, TES, and SJ. Wobble thresholds are given in integer values representing base pair distances. These thresholds define the limit between two features (such as TSS) to be considered a matching feature. There are two collapsing modes which are termed capped and non-capped modes. The capped mode requires that all grouped transcript models (mapped reads) have the same number of exons and all their exons have matching start and end sites as per the user defined wobble thresholds. Matches are performed pairwise in a non-stochastic algorithm. This pair-wise matching is what leads to wobble walking.

The splice junction ranking and local density error algorithm are designed to identify the most likely real splice junctions given a group of matching transcript models. Both the splice junction ranking and LDE rely on user defined threshold of distance from SJ to assess. The LDE feature can be turned on or off. When turned on, the user can specify the distance from the splice junction to assess and the number of allowed mismatches within that distance. If the number of mismatches exceed the threshold, the read is discarded. This is intended to prevent erroneous splice junction predictions. The splice junction ranking can be turned on or off by the user. When turned off, the splice junctions are selected based on the the highest read coverage. When splice junction ranking is turned on, TAMA Collapse ranks the splice junction read support based on the amoun of mismatches flanking the splice junctions. In this method, a splice junction with read support where there are no mismatches flanking the splice junction is given the highest rank and chosen as the final predicted splice junction.

While TAMA Collapse has multiple file outputs, the main output is a bed12 formatted annotation file containing all non-redundant transcript models.

### TAMA merge

TAMA Merge is designed to remove transcript model redundancy either between multiple input annotations or within a single input annotation. TAMA Merge accepts as input 1 or more annotations in bed12 format. TAMA Merge has multiple output files, however the main output file is an annotation file in bed12 format. TAMA Merge also keeps track of the transcript models and their source annotation which were “merged”. This means that for each transcript model, TAMA Merge provides information on which input annotations had transcripts matching it. TAMA Merge uses the same wobble parameter/algorithm and collapsing modes as TAMA Collapse. However, individual input files can be assigned different collapsing modes. This is useful for merging long read data which is likely to contain 5′ truncated transcript models with a reference annotation. In addition to collapsing mode and wobble thresholds, TAMA Merge allows user to assign priority to different input annotation for features such as TSS, TES, and SJ. For instance, a short read derived annotation can be given priority for SJ, while a long read annotation can be given priority for TSS and TES.

### TAMA read support levels

The tama_read_support_levels.py tool is designed to generate a file that relates each transcript and gene model with the ID’s of reads which were used to generate those models. This can also be thought of as producing read count information for transcripts and genes. The tama_read_support_levels.py tool works on all annotation output files from all TAMA modules as well as on PacBio annotation files. This tool was used to identify reads that were involved in read jumbling.

### TAMA filter fragments

The tama_remove_fragment_models.py tool is used to remove transcript models that appear to be fragments of full length models. The criteria for fragment models is that they contain the same internal exon-intron structure as a transcript that is longer on both the 5′ and 3′ ends. The splice junction wobble can be adjusted by the user.

### TAMA remove single read models

The tama_remove_single_read_models_levels.py tool is used to filter a transcriptome annotation based on the amount of read support for each transcript model. This can be run on either the results of TAMA Collapse or the results of TAMA Merge. When used with TAMA Merge with multiple input annotations, tama_remove_single_read_models_levels.py can filter out models based on the number of supporting sources for each transcript model. When TAMA Merge is used to merge a long read data based annotation with a reference annotation. tama_remove_single_read_models_levels.py can be used to filter out models in the long read annotation that do not match the reference annotation. This is how TAMA performs guided annotation.

### TAMA find model changes

The tama_find_model_changes.py tool is designed to identify reads which have different transcript/gene model assignments between different pipelines. This is referred to as read jumble in this study. This tool takes as input a TAMA Merge annotation which was generated by merging annotations from the 2 pipelines to be compared. This tool also requires a read support file generated by tama_read_support_levels.py. Read jumbles are identified by using the read ID’s and comparing the transcript models they are assigned to within the TAMA Merge annotation file. Any read that supports more than 1 transcript model is considered to be involved in a read jumbling event.

### TAMA ORF/NMD pipeline

The TAMA ORF/NMD pipeline is a method for identifying open reading frames (ORF) from transcript models and relating them to known protein coding genes. Nonsense mediated decay (NMD) product predictions are also made by identifying stop codons which occur 50 bp upstream of a splice junction. The first step of the pipeline is the conversion of the transcript nucleotide sequences into amino acid sequences. This is done by looking for all ORF’s which have a stop codon and selecting the longest ORF’s from each frame (3 forward strand frames). Start codons are not required for an ORF prediction, however, if a start codon is not found, the corresponding ORF is labeled as evidence that the transcript is from a degraded RNA. BlastP is then used to relate the resulting amino acid sequences to a protein database. The ORF from each transcript with the best hit to the database is then selected as the predicted true ORF. Using the ORF information, the transcripts are then labeled with attributes based on the protein hit.

### TAMA degradation signature

The TAMA Degradation Signature (DegSig) score is intended to provide a metric for the relative amount of sequencing reads originating from degraded RNA. The DegSig score is calculated by the following formula:

DegSig = (CT − NT)/CT

Where CT is the number of multi-exon transcript models from genes with more than 1 read support after using TAMA Collapse with the capped mode, and NT is the number of multi-exon transcript models from genes with more than one read support after using TAMA Collapse with the no_cap mode.

### Simulated long read datasets and processing for benchmarking

The simulated PacBio and Nanopore datasets (https://figshare.com/articles/RNA_benchmark_datasets/5360998) were produced in another study [11] using PBSIM [12]. These datasets were also used and described in the Stringtie2 paper [9].

Both datasets were mapped to chromosome 19 of the human reference genome as provided in the simulated dataset. Minimap2 [30] (version 2.15-r915-dirty) with the parameters “--secondary=no -ax splice -uf” was used for mapping. Samtools [31] (version 1.9) was used for all SAM/BAM file handling.

For the TAMA Low processing, TAMA Collapse was used with the parameters “-d merge_dup -x ${capflag} -a 200 -z 200 -sj sj_priority -log log_off -b BAM”. For the TAMA High processing, TAMA Collapse was used with the parameters “-d merge_dup -x no_cap -a 300 -m 20 -z 300 -sj sj_priority -lde 3 -sjt 10 -log log_off -b BAM”. After TAMA Collapse, both TAMA Low and TAMA High shared the same processing with tama_remove_fragment_models.py used with default parameters to remove transcript models that appear to be fragments of longer models. This resulted in the final annotations for both pipelines.

For the TAMA Guided pipeline, the output from the TAMA Low TAMA Collapse run was merged with the reference annotation containing both expressed and non-expressed transcript models using TAMA Merge with “-a 300 -z 300 -m 20 -d merge_dup” parameters. The input filelist.txt file for TAMA Merge set both annotations to capped mode with full priority (1,1,1) given to the reference annotation. The tama_remove_single_read_models_levels.py tool was then used with “-l transcript -k remove_multi -s 2” parameters resulting in the final annotation. The tama_read_support_levels.py tool was used at each step of processing to keep track of read support for each transcript model.

For the Stringtie2 pipeline, Stringtie2 (v2.1.3b) was used with the “-L” parameter after mapping.

For the Stringtie2 Guided pipeline, Stringtie2 (v2.1.3b) with “-L -G <reference annotation>” parameters was used. The reference annotation used was the same annotation as used in in TAMA Merge for the TAMA Guided pipeline.

For the TALON pipeline (unguided), a blank database was created using “talon_initialize_database” with default settings and an empty GFF file. Then “talon_label_reads” was used with “--t 1 --ar 20 --deleteTmp” parameters. Then default “talon” was used. This was followed by “talon_filter_transcripts” using “--maxFracA 0.5 --minCount 5 --minDatasets 1” parameters. The default “talon_create_GTF” was used to create a GTF file for the annotation.

For the TALON guided pipeline, a database was created using “talon_initialize_database” with default settings and the same GFF reference annotation file used for TAMA Guided and Striingtie2 Guided. Then default “talon” was used. This was followed by “talon_filter_transcripts” using” --maxFracA 0.5 --minCount 1 --minDatasets 2″ parameters. The default “talon_create_GTF” was used to create a GTF file for the annotation.

All resulting annotations were compared to the annotation file containing all expressed transcript models using GffCompare (v0.11.2).

### Universal human reference RNA and PacBio sequencing

RNA and cDNA library preparation and sequencing were undertaken by Pacific Biosciences. Pacific Biosciences made the data available for public use via a Github repository (https://github.com/PacificBiosciences/DevNet/wiki/Sequel-II-System-Data-Release:-Universal-Human-Reference-(UHR)-Iso-Seq). The RNA library was first created by pooling the Universal Human Reference RNA (Agilent) with SIRV Isoform Mix E0 (Lexogen). cDNA was prepared from the RNA using the Clontech SMARTer kit. The sequencing library was prepared using the Iso-Seq Template Preparation for Sequel Systems (PN 101–070-200) and Sequencing Sequel System II with “Early Access” binding kit (101–490-800) and chemistry (101–490-900). The sequencing library was sequenced on two Sequel II SMRT cells.

### Iso-Seq processing

The UHRR Sequel II Iso-Seq data was processed into CCS reads using the *ccs* tool with the parameters *“--noPolish --minPasses = 1*”. CCS reads with cDNA primers and polyA tails were identified as full-length, non-concatemer (FLNC) reads using *lima (−-isoseq –dump-clips*) and *isoseq3 refine (−-require-polya*).

### Lexogen SIRV Iso-Seq dataset benchmarking

The UHRR Sequel II Iso-Seq data also contained a spike-in of Lexogen SIRV RNA. For the Cupcake pipeline we used the FLNC reads from each SMRT cell and used Cluster/Polish for long read inter-read error correction. We then mapped the resulting reads using Minimap2 (−-secondary = no -ax splice -uf -C5) to the “SIRV_isoforms_multi-fasta_170612a.fasta” reference genome assembly provided by Lexogen. After mapping we ran Cupcake Collapse “collapse_isoforms_by_sam.py” with the Cupcake manual recommended settings “--dun-merge-5-shorter”. We then used Cupcake “chain_samples.py” to merge the assemblies from each SMRT Cell. This resulted in the final annotation for the Cupcake pipeline.

For all the other pipelines (TAMA Low, TAMA High, TAMA Guided, Stringtie2, Stringtie2 Guided, TALON, and TALON Guided), we mapped the FLNC reads to the same reference genome as above using the same parameters for Minimap2.

For the TAMA Low processing, TAMA Collapse was used with the parameters “-d merge_dup -x no_cap -sj sj_priority -log log_off -b BAM -lde 5 -sjt 20 -a 100 -z 100”. For the TAMA High processing, TAMA Collapse was used with the parameters “-d merge_dup -x no_cap -sj sj_priority -log log_off -b BAM -lde 1 -sjt 20 -a 100 -z 100”. After TAMA Collapse, both the TAMA Low and TAMA High pipelines used TAMA Merge (−a 100 -z 100 -d merge_dup) was used to merge the TAMA Collapse outputs from each SMRT Cell. The tama_remove_single_read_models_levels.py tool was then used with “-l transcript -k remove_multi -s 2” parameters resulting in the final annotation. The tama_read_support_levels.py tool was used at each step of processing to keep track of read support for each transcript model.

For the TAMA Guided pipeline, TAMA Collapse (−d merge_dup -x capped -sj sj_priority -log log_off -b BAM -a 0 -m 0 -z 0) was used on the Minimap2 output files for each SMRT Cell. TAMA Merge (−d merge_dup -a 0 -m 0 -z 0) was then used to combined the TAMA Collapse outputs from each SMRT cell. TAMA Merge (−d merge_dup -a 0 -m 0 -z 0) was then used again to match the output with the SIRV annotation file (SIRV_isoforms_multi-fasta-annotation_C_170612a.gtf).. The tama_remove_single_read_models_levels.py tool was then used with “-l transcript -k remove_multi -s 2” parameters resulting in the final annotation. The tama_read_support_levels.py tool was used at each step of processing to keep track of read support for each transcript model.

For the Stringtie2 pipeline, Stringtie2 (v2.1.3b) was used with the “-L” parameter after mapping.

For the Stringtie2 Guided pipeline, Stringtie2 (v2.1.3b) with “-L -G <reference annotation>” parameters was used. The reference annotation used was the same annotation as used in in TAMA Merge for the TAMA Guided pipeline.

For the TALON pipeline (unguided), a blank database was created using “talon_initialize_database” with default settings and an empty GFF file. Then “talon_label_reads” was used with “--t 1 --ar 20 --deleteTmp” parameters. Then default “talon” was used. This was followed by “talon_filter_transcripts” using “--maxFracA 0.5 --minCount 10 --minDatasets 2” parameters. The default “talon_create_GTF” was used to create a GTF file for the annotation.

For the TALON guided pipeline, a database was created using “talon_initialize_database” with default settings and the same GFF reference annotation file used for TAMA Guided and Striingtie2 Guided. Then default “talon” was used. This was followed by “talon_filter_transcripts” using” --maxFracA 0.5 --minCount 5 --minDatasets 2″ parameters. The default “talon_create_GTF” was used to create a GTF file for the annotation.

All resulting annotations were compared to the Lexogen SIRV annotation file (https://www.lexogen.com/wp-content/uploads/2018/08/SIRV_Set2_Sequences_170612a-ZIP.zip) using GffCompare (v0.11.2).

### Chicken brain RNA and PacBio sequencing

The non-cap selected chicken brain Iso-Seq data is from the European Nucleotide Archive submission PRJEB13246 which was previously analyzed and published [4].

The cap selected chicken brain Iso-Seq data was from an adult Advanced Intercross Line chicken whole brain sample. The RNA was extracted from the tissue sample using the Qiagen RNeasy Mini Kit. The RNA was converted to cDNA using the Lexogen TeloPrime kit. The resulting cDNA library was sent to Edinburgh Genomics for sequencing on the Sequel system using 2.0 chemistry.

### TAMA low pipeline for UHRR

Full descriptions of the TAMA algorithms can be found in the wiki pages of the Github repository (https://github.com/GenomeRIK/tama/wiki). FLNC reads were mapped to GRCh38 (Homo_sapiens.GRCh38.dna_sm.primary_assembly.fa) using Minimap2 *(−-secondary = no -ax splice -uf -C5 -t 8*). The resulting bam files were then split into 12 smaller bam files using *tama_mapped_sam_splitter.py* which splits bam files by chromosome thus preventing splitting between reads from the same gene. Split bam files were annotated using *TAMA collapse (−d merge_dup -x no_cap -a 100 -z 100 -sj sj_priority -lde 5 -sjt 20 -log log_off)* then merged into a single bed file using *TAMA merge (−a 100 -z 100*). The tama_read_support_levels.py tool was used at each step of processing to keep track of read support for each transcript model.

### TAMA high pipeline for UHRR

*TAMA collapse* was run on the split bam files using more stringent parameters that filter out any mapped read with more than 1 error within 20 bp of a splice junction (*−d merge_dup -x no_cap -a 100 -z 100 -sj sj_priority -lde 1 -sjt 20 -log log_off*). Merging was done in the same manner as the TAMA Low pipeline. Transcript models supported only by reads from a single SMRT Cell were filtered out using *tama_remove_single_read_models_levels.py (−l transcript -k remove_multi -s 2*). The tama_read_support_levels.py tool was used at each step of processing to keep track of read support for each transcript model.

### Polish pipeline for UHRR

FLNC reads from the *isoseq3 refine* step were clustered using *isoseq3 cluster* and *isoseq3 polish* with default parameters. The output high-quality transcripts were mapped to the genome using Minimap2 *(−-secondary = no -ax splice -uf -C5 -t 8*) and processed using *TAMA collapse (−d merge_dup -x no_cap -a 100 -z 100 -sj sj_priority -lde 5 -sjt 20 -log log_off*). The tama_read_support_levels.py tool was used at each step of processing to keep track of read support for each transcript model.

### Lordec pipeline for UHRR

FLNC reads from the *isoseq3 refine* step were error corrected using LoRDEC *(−k 31 -s 3*) with short read RNA-seq data from the Universal Human Reference RNA (Agilent) (https://www.ncbi.nlm.nih.gov/sra/SRX1426160) (https://rnajournal.cshlp.org/content/22/4/597.full.pdf). The resulting error-corrected reads were processed in the same way as the TAMA Low starting from the mapping step. The tama_read_support_levels.py tool was used at each step of processing to keep track of read support for each transcript model.

### Finding transcript matches and loci overlap between Iso-Seq annotations and the Ensembl annotation

We used TAMA Merge to compare the annotations from each Iso-Seq pipeline (TAMA Low, TAMA High, Polish, and Lordec) to the Ensembl v94 annotation. All input annotations were set to capped mode in the input fielist.txt files. The “-a 300 -z 300 -m 0 -d merge_dup” parameters were used to run TAMA Merge. Transcript matches were identified from the trans_report.txt file while gene loci overlap was identifed from the gene_report.txt file.

### Comparing 5′ completeness between the TAMA high, polish, and Ensembl v94 annotations

We used TAMA Merge to compare the annotations for pairs of annotations (TAMA High-Polish, TAMA High-Ensembl, Polish-Ensembl). Both annotations in each merging were given no_cap parameters in the filelist.txt input file. We used the same TAMA Merge settings as were used for identifying matching transcript models between annotations. We used the TAMA Merge trans_report.txt output file to identify which source annotation had the longer 5′ representation for each matching transcript model.

### Degradation signature analysis

We split the SAM files from the mapping by chromosome. We then used these single chomosome SAM files as inputs to 2 TAMA Collapse runs. One TAMA Collapse run used the capped mode and the other run used the no_cap mode. Both runs used “-a 100 -z 100 -sj sj_priority -lde 5 -sjt 20 -log log_off -b BAM” parameter settings. We then used the trans_read.bed files from each pair of TAMA Collapse runs as inputs for the tama_degradation_signature.py tool which calculated the DegSig scores.

### Mismatch and wobble analysis

The mismatch profiles for the mapped FLNC, Cluster/Polish corrected, and LoRDEC corrected reads were extracted from the TAMA Collapse read.txt output files generated in each pipeline.

To assess the wobble between each pipeline and the Ensembl annotation, we used *TAMA merge* with parameter settings *(−a 300 -z 300 -m 30 -d merge_dup*) which considers any transcripts which have up to 300 bp difference in their transcription start and end and up to 30 bp difference in their splice junctions starts and ends to have “nearly identical structures”. This is the definition for matching at transcript level.

### Read jumbling analysis

Read ID’s were tracked through each processing step using the tama_read_support_levels.py tool. TAMA Merge was used to combine the annotations from the different pipelines (TAMA Low-Polish, TAMA Low-Lordec) using the same parameters as the was used in the wobble analysis. The TAMA Merge output and tama_read_support_levels.py outputs were used as input for the tama_find_model_changes.py tool that identified reads which had different transcript model assignment between each pair of pipelines.

### Coding potential analysis

For the Ensembl match evidence of coding potential, we labelled the Iso-Seq annotation genes as coding if they had any overlap on the same strand as an Ensembl-annotated protein coding gene.

CPAT was used with default parameters and the built-in Human Hex models. A cutoff score of 0.364 (suggested by the CPAT creators [27]) was used to segregate between coding and non-coding transcripts.

We used the TAMA ORF/NMD pipeline for the third source of coding evidence. The transcript models were converted into fasta sequences using Bedtools [32]. ORFs were predicted for each transcript from the fasta file then translated into amino acid sequences. BlastP [33] (*−evalue 1e-10 -ungapped -comp_based_stats F*) was used to match the amino acid sequences to the UniRef90 database, where the top hits were selected as the best ORF prediction. Transcripts with no hits were considered to be non-coding.

### Matching TAMA high annotation to Ensembl v100

For identifying gene models found in the Ensembl v100 human annotation matching gene models predicted in the TAMA High annotation which were not present in the Ensembl v94 human annotation, we used TAMA Merge with “-m 0 -a 300 -z 300” parameters in capped mode for all three annotations (TAMA High, Ensembl v94, and Ensembl v100). These parameters group transcript models between the annotations if they share the exact same splice junctions and exon chaining but with an allowance of up to 300 bp difference in TSS and TES. We then identified all gene models which were the product of merging a TAMA High annotation gene with an Ensembl v100 gene and with no Ensembl v94 gene represented for that loci.

## Supplementary information

**Additional file 1:** **Figure S1.** Histogram plot of FLNC read lengths.

**Additional file 2:** **Table S1.** Table of long read datasets information on number of mapped reads, reference annotation, and genome assembly scaffold numbers.

## Data Availability

The PacBio Universal Human Reference RNA Sequel II Iso-Seq dataset is available from https://github.com/PacificBiosciences/DevNet/wiki/Sequel-II-System-Data-Release:-Universal-Human-Reference-(UHR)-Iso-Seq. The short read Illumina RNA-seq data used for LoRDEC error correction are available in the National Center for Biotechnology Information Sequence Read Archive under accession number SRP066009 (https://www.ncbi.nlm.nih.gov/sra/SRX1426160). The non-cap selected chicken brain Iso-Seq data is available from the European Nucleotide Archive under accession number PRJEB13246. The TeloPrime cap selected chicken brain Iso-Seq data is available from the European Nucleotide Archive under accession number PRJEB25416. The simulated long read dataset is available here: https://figshare.com/articles/RNA_benchmark_datasets/5360998

## Abbreviations

CCS: Circular consensus sequence
DegSig: Degradation signature
ECC: Exon cascade collapse
FLNC: Full length non-chimeric reads
LDE: Local density error
lncRNA: Long non-coding RNA
NMD: Nonsense mediated decay
PacBio: Pacific bioscience
RNA-seq: RNA sequencing
SIRV: Spike-in RNA variant control
TAMA: Tranascriptome annotation by modular algorithms
TSSC: Transcription start site collapse
UHRR: Universal human reference RNA
