# Supplementary figures and images for: Illuminating the dark side of the human transcriptome with long read transcript sequencing
Source: BMC Genomics. 2020 Oct 30;21:751. doi: 10.1186/s12864-020-07123-7 (PMC7596999; doi:10.1186/s12864-020-07123-7)

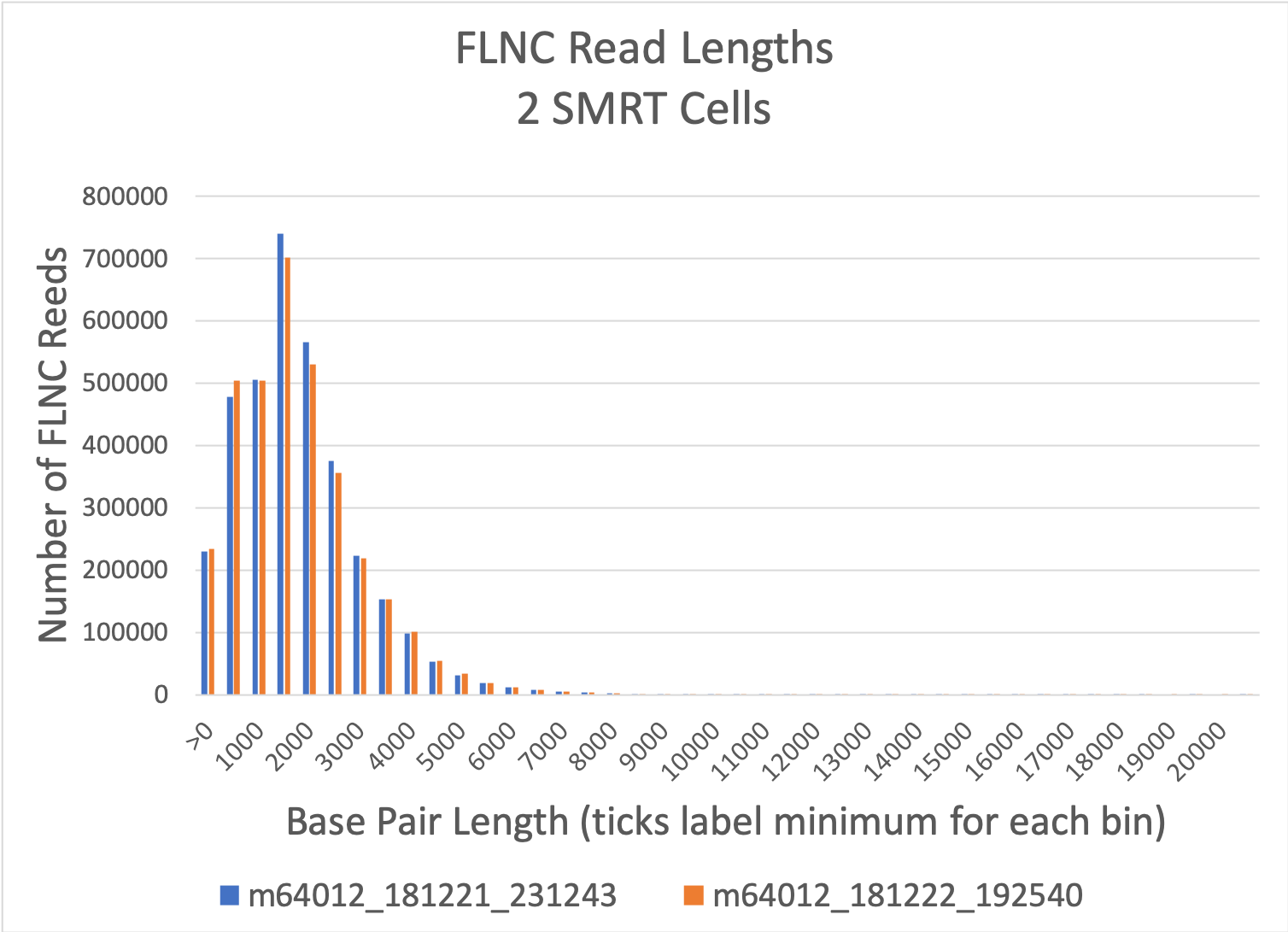

Supplement: Supplementary file 1 — Additional file 1: Figure S1. Histogram plot of FLNC read lengths. [file 12864_2020_7123_MOESM1_ESM.png]
