# Supplementary material for: Illuminating the dark side of the human transcriptome with long read transcript sequencing
Source: BMC Genomics. 2020 Oct 30;21:751. doi: 10.1186/s12864-020-07123-7 (PMC7596999; doi:10.1186/s12864-020-07123-7)
Supplement: Supplementary file 2 — Additional file 2: Table S1. Table of long read datasets information on number of mapped reads, reference annotation, and genome assembly scaffold numbers. [file 12864_2020_7123_MOESM2_ESM.docx]

**Supplementary Table 1 Summary of long read RNA sequencing datasets**

|  | Mapped Reads | Reference  Annotation Genes | Reference Annotation Transcripts | Annotated Reference Scaffolds |
| --- | --- | --- | --- | --- |
| PacBio Simulated | 77,973 | 2,728 | 14,108 | 1 |
| Nanopore Simulated | 73,144 | 2,728 | 14,108 | 1 |
| SIRV Iso-Seq | 114,543 | 18 | 69 | 7 |
| UHRR Iso-Seq | 6,578,644 | 58,735 | 206,601 | 47 |
